# A functional map of NFκB signaling identifies novel modulators and multiple system controls

**DOI:** 10.1186/gb-2007-8-6-r104

**Published:** 2007-06-06

**Authors:** Thomas A Halsey, Longlong Yang, John R Walker, John B Hogenesch, Russell S Thomas

**Affiliations:** 1 The Hamner Institutes for Health Sciences, 6 Davis Drive, PO Box 12137, Research Triangle Park, NC 27709-2137, USA; 2 Genomics Institute of the Novartis Research Foundation, 10675 John J. Hopkins Drive, San Diego, CA 92121, USA; 3 Institute for Translational Medicine and Therapeutics, 810 Biomedical Research Building, University of Pennsylvania School of Medicine, 421 Curie Boulevard, Philadelphia, PA 19104-6160, USA; 4 Almac Diagnostics, 801-1 Capitola Drive, Durham, NC 27713, USA

## Abstract

**Background:**

The network of signaling pathways that leads to activation of the NFκB transcription factors is a branched structure with different inputs and cross-coupling with other signaling pathways. How these signals are integrated to produce specific, yet diverse responses is not clearly understood. To identify the components and structural features of the NFκB network, a series of cell-based, genomic screens was performed using a library of approximately 14,500 full-length genes.

**Results:**

A total of 154 positive and 88 negative modulators of NFκB signaling were identified. Using a series of dominant-negative constructs and functional assays, these modulators were mapped to the known NFκB signaling cascade. Most of the positive modulators acted upstream of the IκB kinase complex, supporting previous observations that the IκB kinases represent the primary point of convergence in the network. A number of negative modulators were localized downstream of the IκB kinase β (IKBKB) subunit, suggesting that they form an additional layer of negative control within the system. The expression of the modulators at the RNA level was distributed disproportionately across tissues, providing flexibility in network structure, and the number of positive and negative modulators present in a given tissue was highly correlated, suggesting that positive and negative regulation is balanced at the tissue level.

**Conclusion:**

The relative locations of the modulators are consistent with an hourglass structure for the NFκB network that is characteristic of robust systems. The tissue distribution of the modulators and downstream location of the negative modulators serve as layers of control within the system that allow differential responses to different stimuli.

## Abstract 2

Using cell-based genomic screens and functional assays, positive and negative modulators of NFκB signaling were identified and mapped onto the known NFκB signaling cascade.

## Background

The NFκB transcription factor represents a collection of dimeric complexes from the NFκB/Rel family. The transcriptional complexes regulate a broad spectrum of genes that function in a variety of key biological processes [1]. At the network level, the NFκB signaling pathway is a branched structure with a variety of inputs that include proinflammatory cytokines, T- and B-cell receptors, growth factors, UV radiation, and pathogen-associated signals (for example, bacterial lipopolysaccharide (LPS)). The various branches within the NFκB pathway are all associated with different cascades of signaling events that eventually converge at a core set of IκB kinases (IKKs). Historically, the branches have been organized into canonical and atypical classes. Among the branches in the canonical class, stimulation by ligands such as tumor necrosis factor-α (TNF), interleukin-1 (IL1), or LPS leads to signaling events that activate the IκB kinase (IKK) complex containing the CHUK (IKKα) and IKBKB (IKKβ) catalytic subunits along with the IKBKG (IKKγ) regulatory subunit [2]. The activated IKK complex phosphorylates the inhibitory IκB proteins, leading to their ubiquitination and degradation by the 26S proteosome [2]. In the branches classified as atypical, stimulation is limited to a smaller subset of NFκB activators such as LTB and TNFSF13B. Signal initiation is followed by a series of signaling events that activate CHUK homodimers [3,4]. The CHUK homodimers phosphorylate the inhibitory NFKB2 precursor, leading to processing into active NFKB2 and dimerization with RELB [3-5].

Despite the breadth of knowledge about NFκB signaling, details are still being discovered about how other signaling pathways interconnect within the greater NFκB network and how various signaling inputs are integrated to form different outputs. A variety of signaling pathways and subsystems are known to cross-couple with the NFκB pathway at different nodal points and modulate NFκB signaling. Examples of cross-coupling include AP-1 [6], HSF1 [7], γ-interferon [8], small GTPases [9], and PI3K [9]. The prevalence of cross-coupling, together with the branched structure of the NFκB network, results in a complex, interconnecting system whose structure is both context and tissue dependent. A previous study of the Toll-like receptor (TLR) branch of the NFκB network suggested that the extent of cross-coupling within this network provides the capability for the system to generate differential responses to different stimuli [9].

To uncover the various genes and structural features involved in the extended NFκB signaling network, several research groups have applied advanced genomic and proteomic technologies. In one study, a large-scale full-length gene screen was performed to identify activators of the NFκB and MAP kinase (MAPK) pathways [10]. The investigators showed that a significant number of activators were shared between the two pathways [10]. Other studies have utilized RNA interference [11,12] and protein-interaction measurements [12] to identify functional and physical modulators of NFκB. In the present study, the greater NFκB signaling network was experimentally interrogated using a series of high-coverage gain-of-function and loss-of-function genomic screens containing 14,500 full-length mouse and human genes. The genomic screens were used to comprehensively identify both positive and negative modulators of the NFκB pathway. Following the initial screens, the positive and negative modulators were mapped to specific locations in the NFκB pathway by screening them in tandem with a series of dominant-negative and constitutively active mutants of key NFκB regulators. The relative placement of the modulators within the network and their distribution across tissues were used to identify the multiple system controls within the NFκB network that ultimately influence the specificity and diversity of the response.

## Results

### Identification of positive NFκB modulators using gain-of-function full-length gene screens

**Figure 1:**
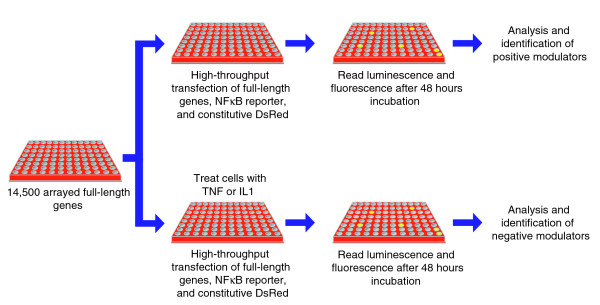
Flowchart outlining the steps in the gain-of-function and loss-of-function genomic screens. The gain-of-function screen was used to identify positive modifiers of the NFκB signaling pathway and the loss-of-function screen was used to identify negative modifiers. Approximately 14,500 full-length human and mouse genes were screened for activity.

To identify potential positive modulators, approximately 14,500 mouse and human full-length genes were screened for their ability to activate an NFκB luciferase reporter. Individual genes were transfected into HEK-293T cells together with the NFκB reporter and a constitutively expressed DsRed fluorescent protein using high-throughput transfection methods (Figure 1). In the initial screen, 183 genes activated the NFκB reporter greater than three-fold when normalized to the fluorescent transfection control and had luciferase values greater than 5 standard deviations (SD) from the experimental mean. These genes were rearrayed and screened in triplicate to confirm activation of the NFκB reporter. Eighty-four percent (154 genes) of the original positive modulators were confirmed in these studies on the basis of an average three-fold activation and an adjusted *p *value of less than 0.05. Of these 154 genes, 45 have been previously shown to modulate NFκB signaling (Additional data file 1).

**Figure 2:**
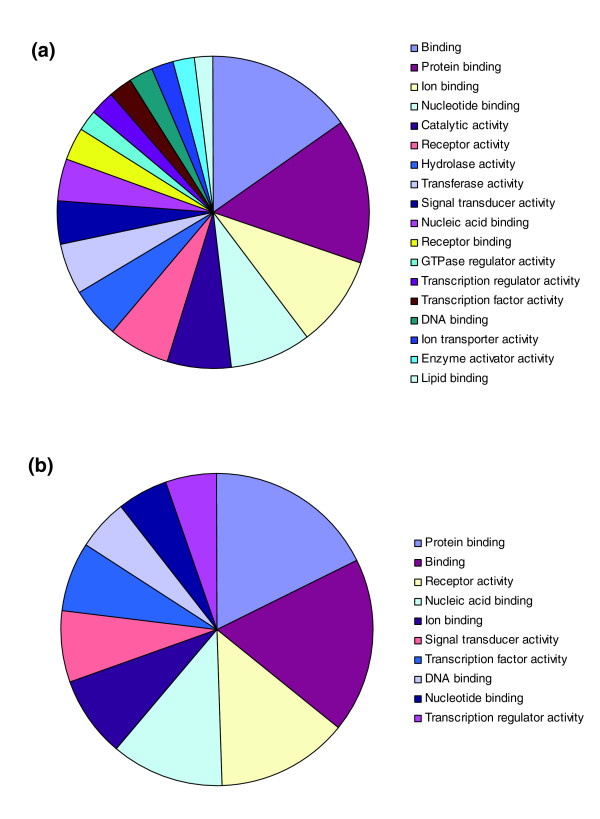
Functional classification of the NFκB modulators identified in the functional genomic screens. Functional classification was performed using NIH David 2.1. **(a) **Classification of the positive modulators. **(b) **Classification of the negative modulators.

Among the known NFκB modulators, genes within both the canonical and atypical NFκB signaling branches were identified, including known receptor ligands (*TNF*, *IL1B*, and *LTA*), TNF receptors (*TNFRSF1A*, *TNFRSF10A*, *TNFRSF11A*, *TNFRSF12A*, *TNFRSF25*, and *TNFRSF10B*), B-cell receptor (*CD40*), adaptor proteins (*TIRAP*, *MYD88*, and *FADD*), TNF receptor-associated factors (*TRAF2 *and *TRAF5*), members of the NFκB transcriptional complex (*RELA*, *RELB*, and *NFKB1*), and others. The known positive modulators were mapped to the canonical TNF, IL1, B-cell receptor, and T-cell receptor signaling pathways (Additional data files 5 and 6). The functional breakdown of the positive modulators is shown in Figure 2a. The breakdown contains a diverse array of categories that reflect the wide range of stimuli capable of activating NFκB and potential cross-talk with other signaling pathways.

### Identification of negative NFκB modulators using loss-of-function full-length gene screens

In order to identify inhibitors of NFκB activity, full-length cDNAs were introduced into HEK-293T cells as described in the preceding section and the cells were incubated in the presence of either TNF or IL1 (Figure 1). A total of 235 genes were identified that reduced NFκB reporter activity greater than three-fold when normalized to the fluorescent transfection control and had luciferase values less than 5 SD from the experimental mean. These genes were rearrayed and screened in replicate to confirm the reduction of the NFκB reporter activity when exposed to either TNF or IL1. Forty-four percent (104 genes) of the original negative modulators were confirmed in these studies based on an average three-fold reduction and an adjusted *p *value of less than 0.05 (Additional data file 2). To exclude nonspecific negative modulators, an identical assay was performed using the glucocorticoid response element as the reporter and dexamethasone as the stimulus. Sixteen genes that showed inhibition of reporter activity in both screens were labeled as 'general inhibitors' and were removed from subsequent analysis. For the remaining 88 negative modulators, the functional breakdown is shown in Figure 2b. The breakdown was less diverse than with the positive modulators and had a relatively high percentage of genes related to DNA binding and transcriptional activity, suggesting that a number of the negative modulators act downstream in the pathway.

Among the 88 negative modulators, 16 have been previously shown to negatively regulate the NFκB pathway; they include genes such as *NFKBIB *(IκBβ) [2], *NFKBIE *(IκBε) [2], *PIAS4 *[13], and *RHOB *[14]. The known negative modulators were also mapped to the canonical TNF, IL1, B-cell receptor, and T-cell receptor signaling pathways (see Additional data files 5 and 6). Several genes identified as negative modulators in our screen have been previously shown to positively regulate NFκB and three genes (*GPD1*, *TRAF2*, and *TSPAN13*) were identified as both positive and negative modulators in our screens (see Additional data files 1 and 2). For most of the genes previously shown to be positive regulators, it is unclear why they are negative modulators in our system. One possibility is simple methodological differences. For example, some genes previously shown to positively modulate NFκB were identified in a screen performed 24 hours after transfection [10], whereas genes in our study were identified 48 hours after transfection and in the presence of TNF or IL1. A more likely possibility is that they can either negatively or positively modulate NFκB, depending on the cellular context. There are numerous examples of differential gene function depending on cellular context [15-19], and this behavior has also been observed for genes within the NFκB network [20,21]. For example, TRAF2 has been previously shown to play a positive role in CD40 signaling in B cells and a negative role in TNF signaling in macrophages [21]. The mechanism underlying these different behaviors has not been conclusively identified, but it has been suggested that it can both activate and degrade proteins by attaching distinct types of polyubiquitin chains [22]. In addition, activation of the IKK complex can have pro-apoptotic or anti-apoptotic effects, depending on timing and mechanism of activation [20]. Therefore, it is possible that a cell-based, genomic screen would identify differential behaviors for true modulators of NFκB under different treatment conditions (that is, untreated cells to identify positive modulators and cells treated with TNF or IL1 to identify negative modulators).

### Tissue expression of the NFκB modulators

**Figure 3:**
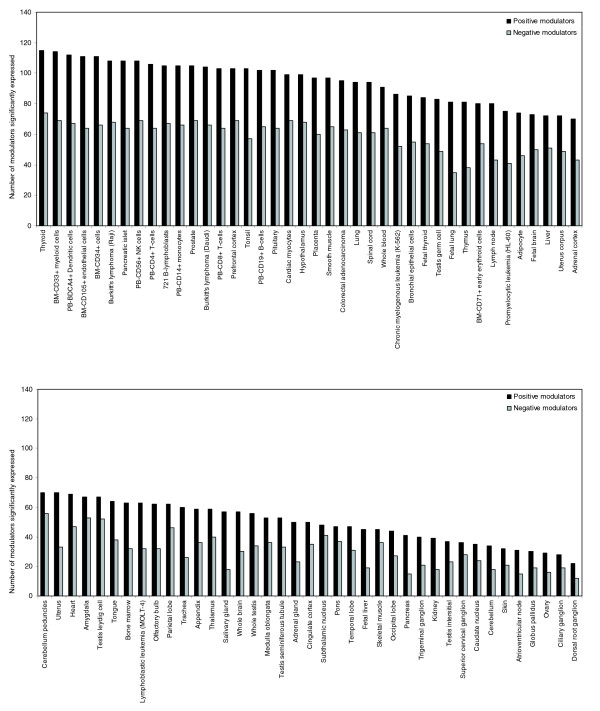
Tissue expression of positive and negative NFκB modulators. Gene-expression data for 79 human tissues using the human Affymetrix U133A array was obtained from Symatlas [23]. The mean expression level and a 99% confidence interval for each gene were then calculated across all 79 human tissues. Using the lower 99% confidence limit as a cutoff, modulators that fell below the cutoff were considered absent in that tissue while those that were expressed above the cutoff were considered present. The black bars represent the number of positive modulators present in a given tissue out of 131 that were contained on the microarray. The light-gray bars represent the number of negative modulators present in a given tissue out of 80 that were contained on the microarray. The number of positive and negative modulators significantly expressed in each tissue was significantly correlated (*r *= 0.944, *p *< 0.001).

To obtain a general understanding of how the positive and negative NFκB modulators were co-expressed across various tissues, gene-expression data from 79 different tissues was obtained from Symatlas [23]. The mean expression level and a 99% confidence interval for each modulator were then calculated across the 79 human tissues. Using the lower 99% confidence limit as a cutoff, modulators that fell below the cutoff were considered absent in that tissue, whereas those that were expressed above the cutoff were considered present. As expected, tissues involved in the immune response (for example, peripheral blood BDCA4^+ ^dendritic cells) had a higher average number of positive and negative modulators present in the tissue when compared with non-immune tissue (152 versus 108; *p *< 0.0001) (Figure 3). These results suggest that regulation by these modulators is not distributed uniformly across tissues and that one mechanism for controlling the number of branches in the NFκB network is through differential expression. This mechanism has been described previously for G-protein-coupled receptors [24]. Notably, the number of positive and negative modulators that were present in a given tissue was highly correlated (*r *= 0.944; *p *< 0.001) suggesting that the degree of positive regulation is counterbalanced by negative regulators at the tissue level.

### Contextual organization of the positive NFκB modulators

**Figure 4:**
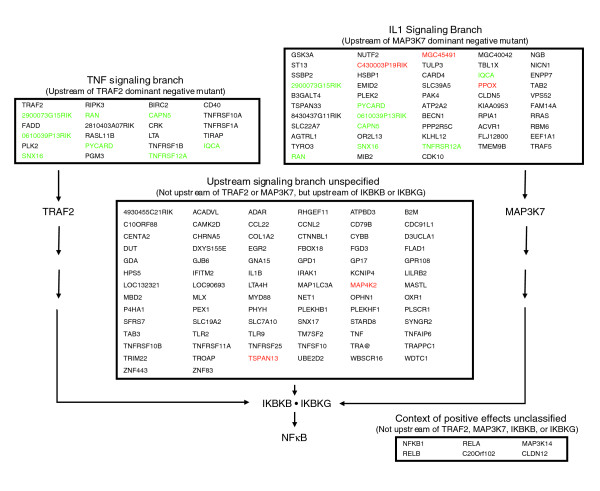
Contextual organization of the NFκB positive modulators. Organization was determined on the basis of follow-up screens with a series of dominant-negative mutants (TRAF2, MAP3K7, IKBKB, and IKBKG). If activation of the NFκB reporter was blocked by the dominant-negative mutant (≥ 70% average reduction), the positive modulator was considered upstream of the dominant-negative mutant. In the boxes upstream of TRAF2 and MAP3K7, green text identifies genes that were inhibited by both the TRAF2 and MAP3K7 dominant-negative mutants. In the boxes upstream of MAP3K7 and the IKK complex, red text identifies genes that were inhibited only by the IKBKB mutant. Genes identified as IKBKB specific showed ≥ 70% inhibition by the IKBKB dominant-negative mutant and <70% inhibition by the IKBKG dominant-negative mutant. In addition, IKBKB-specific genes were required to show a fivefold greater reduction in NFκB activation by the IKBKB dominant-negative mutant as compared with the IKBKG mutant. Those genes that were inhibited by both the IKBKB and IKBKG dominant-negative mutants were shown in black text. Genes that were not significantly inhibited by any of the dominant-negative mutants are listed in the lower right-hand box and were presumed to act downstream in the pathway or through an independent branch.

To map the positive modulators within the NFκB structural network, the effects of each modulator was examined in the presence of a series of dominant-negative mutants. These mutants were chosen due to their roles and relative locations within the NFκB network. Individual positive modulators were screened in triplicate with IKBKB, IKBKG, TRAF2, and MAP3K7 (TAK1) dominant-negative mutants (Additional data file 3). If the activation of the NFκB reporter was blocked by the dominant-negative mutant (≥ 70% average reduction), the positive modulator was considered upstream of the dominant-negative mutant. The results from this analysis organized the positive modulators into four distinct groups - upstream of TRAF2, upstream of MAP3K7, upstream of the IKK complex, and no inhibition by any dominant-negative mutant (Figure 4).

The group of positive modulators identified as upstream of TRAF2 included a variety of genes with a known dependence on TRAF2 for signal propagation. They included genes for several TNF receptors (for example, *TNFRSF1A*, *TNFRSF1B*, and *TNFRSF12A*), and for *LTA*, *FADD*, *BIRC2*, *RIPK3*, and *CD40*. Of these genes, the FADD protein is known be a part of a complex that includes TRAF2, RIPK1, and TRADD [25]. For *BIRC2*, physical interaction with TRAF2 alters nuclear translocation of the protein [26] and a dominant-negative TRAF2 has been demonstrated to inhibit RIPK3 signaling [27]. Other genes in this group have been associated with NFκB signaling, but have not previously been identified as TRAF2-dependent. These included *PYCARD*, *TMEM9B*, and *TIRAP *[10,28,29]. For *TIRAP*, previous reports have shown that it is involved in Toll-like receptor signaling [29], raising questions about its dependence on TRAF2. One explanation is that the overexpression of the TRAF2 dominant-negative mutant results in a nonspecific inhibition of TRAF6. However, neither *MYD88 *nor any of the Toll-like receptors that were also identified as positive modulators were inhibited by the overexpression of the TRAF2 mutant. Another explanation is that the physical association identified between TBK1 and TIRAP [30] is part of a larger complex that also includes TRAF2. TRAF2 has also been shown to associate with TBK1 [31] and formation of the larger complex may be required for TIRAP signaling.

In the group of positive modulators identified as acting upstream of MAP3K7, several genes have a known dependence on MAP3K7 for signaling, including *MAP3K7IP2 *and *CARD4 *(Figure 4). Of the proteins encoded by these genes, MAP3K7IP2 activates MAP3K7 by forming a complex with TRAF6, MAP3K7 and TAB1 [32]. CARD4 has been shown to induce transcription of *MAP3K7 *and of the genes for other components of the IL1/TLR branch leading to activation of NFκB [33]. Other genes in this group have been associated with NFκB signaling, but not previously identified as MAP3K7-dependent. These included *PAK4*, *NUTF2*, *RRAS*, *EEF1A1*, *TRAF5*, *TBL1X*, *PYCARD*, *ATP2A2*, *TMEM9B*, *TRAF2*, and *TNFRSF12A*. For *TRAF2 *and *TRAF5*, previous reports have linked their involvement with the TNF signaling branch [34,35]. However, other reports have shown that TRAF2 can activate MAP3K7 under certain conditions [12,36] and that MAP3K7 still contributes to IκB phosphorylation under TNF stimulation [37], suggesting that signaling through each branch is not exclusive. This cross-talk may contribute to the number of genes that were found to be upstream of both TRAF2 and MAP3K7 (Figure 4, green text).

For the positive modulators inhibited by either the TRAF2 or MAP3K7 dominant-negative mutant, all genes were also inhibited by at least one of the IKK mutants. For the genes identified as upstream of TRAF2, MAP3K7, and the IKK complex, the majority was inhibited by both the IKBKB and IKBKG dominant-negative mutants, and all genes required IKBKB for activation. This suggests that most of the positive modulators were from what has historically been referred to as the canonical branch. Interestingly, five positive modulators were not inhibited by the IKBKG dominant-negative mutant: they comprise three of the genes upstream of MAP3K7 and two of the genes upstream of the IKK complex (Figure 4, red type). The IKBKG dominant-negative mutant used in the analysis is deficient for binding to CHUK and IKBKB [38], suggesting that IKBKG binding within the IKK complex is not required for NFκB activation by these genes.

The group of positive modulators identified as upstream of IKBKB and IKBKG was substantially larger than those identified as upstream of TRAF2 and MAP3K7. This primarily reflects its importance as point of convergence in the pathway, as it integrates signals from the various upstream branches [39]. The size of the group may also have been inflated as a result of the relatively strict analysis criteria that may not have correctly identified some genes as TRAF2- or MAP3K7-dependent. These genes include those for the Toll-like receptor *TLR9*, three TNF receptors (*TNFRSF10B*, *TNFRSF11A*, and *TNFRSF25*), and genes such as *TAB3 *and *IL1B*. The signals from these genes were inhibited by the TRAF2 or MAP3K7 dominant-negative mutants, but not to the extent required by the cutoff. Apart from these genes, other positive modulators that are generally accepted to be upstream of TRAF2 or MAP3K7 may have slipped into the IKK group as a result of the branched structure of the network, which would allow the signal to be propagated around the dominant-negative mutant. For example, MYD88 plays a central role in IL1/TLR signaling and has been shown to activate NFκB through both MAP3K7-dependent and -independent mechanisms [9].

In the final group of positive modulators, six genes were not found to be upstream of any of the dominant-negative mutants used in these studies. This group included genes encoding the components of the NFκB transcription complex (*RELA*, *RELB*, and *NFKB1*), an NFκB-inducing kinase (*MAP3K14*), a member of the claudin superfamily (*CLDN12*), and a relatively uncharacterized gene (*C20Orf102*). The contextual placement of *MAP3K14*, *CLDN12*, and *C20Orf102 *was difficult to localize as the lack of inhibition by the dominant-negative mutants could mean that the modulators act through a separate, independent branch that converges at the transcriptional complex. For *MAP3K14*, previous reports are contradictory, with some investigators demonstrating MAP3K14 activation of the IKK complex [40-42] whereas others have reported that MAP3K14 can activate NFκB independently of IKBKG through a p38 MAPK-dependent RELA phosphorylation pathway [43].

### Contextual organization of the negative NFκB modulators

**Figure 5:**
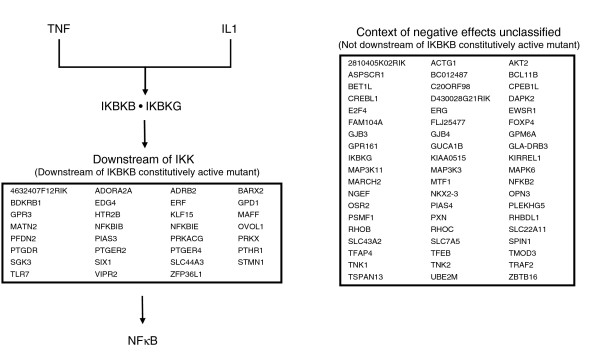
Contextual organization of the NFκB negative modulators. Organization was determined on the basis of follow-up screens with an IKBKB constitutively-active mutant. If activation of the NFκB reporter was blocked by the negative modulator (≥ 70% average reduction), the negative modulator was considered downstream of the constitutively active mutant. If activation of the NFκB reporter was not blocked, the negative modulator was considered unclassified as to its inhibitor influence.

To assess the relative location of the negative modulators, each modulator was screened together with a constitutively active IKBKB mutant (Additional data file 4). If activation of the NFκB reporter was blocked by the negative modulator (≥ 70% reduction), the negative modulator was considered downstream of the constitutively active mutant. If activation of the NFκB reporter was not blocked, the negative modulator was considered unclassified as to its inhibitor influence. For the group of negative modulators downstream of IKBKB, several genes were identified that were previously demonstrated to inhibit NFκB signaling (Figure 5). These genes include *NFKBIB*, *NFKBIE*, *PIAS3*, *PRKACG*, *PTGER2*, and *PTGER4*. The downstream effects of the two IκB genes (*NFKBIB*, *NFKBIE*) have been well established [2], whereas PIAS3 functions downstream by binding to RELA and suppressing NFκB-dependent transcription [44]. The gamma catalytic subunit of protein kinase A (PRKACG) has been shown to repress NFκB signaling by phosphorylating the p50 homodimer, which acts a transcriptional repressor by maintaining DNA binding in unstimulated cells [45]. Finally, for the *PTGER2 *and *PTGER4 *genes, a clear mechanism has not been elucidated, but a recent report by Akaogi and colleagues [46] showed that the NFκB-dependent transcription of TNF was inhibited by 70% in response to activation of prostaglandin E2 and E4 receptors.

### Constructing a signaling network map for the NFκB modulators

To combine the experimental data into a structural network representing NFκB signaling, an adjacency matrix was constructed based on the orientation flags (that is, upstream, downstream, or unclassified) from the dominant-negative and constitutively active screens used in the contextual organization analysis. The adjacency matrix containing the experimental data was then merged with a separate adjacency matrix containing members of the currently accepted NFκB signaling network. By using the known NFκB network as a scaffold, we were able to include additional signaling intermediates and create a more hierarchically consistent network. Based on the combined adjacency matrix, a network map was constructed as a rooted tree with the NFκB complex serving as the obligate root node. The longest path was calculated from each terminal node to the root node using a previously defined algorithm [47]. The inferred NFκB structural network is provided as an additional data file (Additional data file 7).

Constructing the NFκB signaling map provides insight into the potential logic embedded within the network and allows additional hypotheses to be generated regarding influence and potential cross-talk from the positive and negative modulators identified in this investigation. The potential for positive cross-talk in our investigation was concentrated upstream of MAP3K7 and directly upstream of IKBKB and IKBKG. In contrast, a significant number of negative modulators entered the network between the IKK complex and the activated transcription factor, providing a large number of modes for negative regulation.

## Discussion

Technological advances and the improved annotation of the human genome have provided a sound foundation for examining the composition and structure of signaling networks. By building on currently accepted experimental methodologies, we were able to develop a functional genomic strategy for identifying positive and negative modulators of the NFκB signaling network and organize these modulators contextually within the network structure. The NFκB signaling network has been well studied, and many of the network components have been ascribed specific functional roles. Using these previous observations, we were able to both test the validity of our functional genomic approach and identify novel components and structural features of the network.

**Figure 6:**
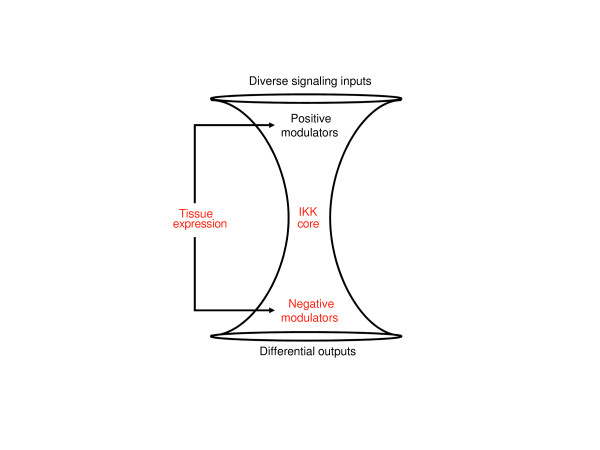
An hourglass model of the NFκB signaling network representing the multiple controls within the system that allow differential responses to diverse stimuli. The hourglass shape is characteristic of many robust systems in biology. At the top of the hourglass, the numerous signaling branches and inputs from cross-talk are funneled into the central core regulated by the IKKs. The IKKs integrate these signals to produce a wide-range of cellular responses that are specific to the stimuli. The specificity and diversity are achieved at multiple levels of regulation within the hourglass model (red text). Tissue expression add or subtract inputs to the model depending on which processes are needed in a given cell type. The IKK core regulates responses based on the type, level, and temporal nature of the activation. Negative regulators downstream of the IKK core represent a third level of control.

The 154 positive modulators and 88 negative modulators that were identified in the functional genomic screens provide a better understanding of the breadth of system controls within the NFκB network. The positive modulators were from a diverse array of functional categories that reflected the wide range of stimuli and cross-coupling with the NFκB network, whereas the negative modulators were less diverse and more focused on downstream processes. Contextual organization of the modulators and generation of a signaling network map showed that most of the positive modulators acted upstream of MAP3K7 and the IKK complex, while fewer were found upstream of TRAF2. This is consistent with a previous study that suggested that the majority of cross-talk interactions within the IL1/TLR branch were concentrated in the region below MYD88 and TRAF6 [9]. Taken together, these results are consistent with previous observations that the three IKKs (CHUK, IKBKB, and IKBKG) represent the point at which the majority of signals converge in the network [39,48] and serve as the core of an hourglass or bow-tie structure that is characteristic of many robust systems [9,49,50] (Figure 6).

Differences in the type, level, and temporal activation of the IKK core is one mechanism that allows the NFκB network to provide a virtual hyperplane of differential responses from a wide range of stimuli. For example, the type of activation of the IKK core through either CHUK homodimers in the atypical branch or through IKBKB in the canonical branch is one mechanism for achieving different outputs from a different set of input signals [2]. In our investigation, we identified a small subset of the positive modulators that did not require IKBKG for their effects. It is possible that activation of the IKK core independent of IKBKG contributes to a different type of output from the NFκB network. Additional research is needed to confirm these observations and understand the context in which this subset of genes participates in NFκB signaling.

In contrast to the positive modulators, a significant number of negative modulators were localized downstream of IKBKB. Notably, these negative modulators inhibited NFκB signaling in the same general location as the IκB proteins. Hoffmann and colleagues [51] showed that the individual IκB proteins function differently in their ability to negatively regulate NFκB signaling. NFKBIA provides strong negative feedback that inhibits short-term NFκB responses, whereas NFKBIB and NFKBIE dampen oscillations in the system and inhibit NFκB during longer stimulations. The temporal differences among these IκB proteins generate specificity at the level of gene activation and provide an additional layer of control within the NFκB network for generating differential output from different inputs. Although we do not know the mechanism of negative regulation for many of the modulators identified in our screen, the fact that they inhibit NFκB signaling downstream of the IKK core is highly suggestive that they also participate as an additional layer of negative control similar to the IκB proteins. The large number of negative modulators in this group suggests that this type of control is a relatively common mechanism within the NFκB network to increase specificity and diversity in the output.

By comparing the expression of the positive and negative modulators across tissues, a third control mechanism was identified. Processes or branches in the network can be added or subtracted on an individual tissue or cell-type basis. In our study, the highest numbers of positive and negative modulators were significantly expressed in immune tissues, underscoring the need to integrate diverse sets of signals in these cells from the various upstream branches into decisions on cell fate, cytokine release, cell adhesion, second messenger release, and others. The number of positive and negative modulators that were significantly expressed in each tissue was highly correlated, suggesting that as the number of upstream branches increases, so must the number of genes in the negative control layer downstream of the IKK core.

## Conclusion

The application of functional genomic tools in this study has identified many potential novel modulators of NFκB signaling as well as provided insights into several structural and regulatory characteristics of the network. The insights build upon the hourglass architecture that has been proposed by Kitano and colleagues for a variety of signaling pathways [49,50,52] and identify additional layers of control for converting a diverse set of input signals to differential outputs (Figure 6). The additional layers consist of a potential upstream branch that does not require IKBKG for NFκB activation, many negative modulators that act downstream of the core IKK complex, and distribution across tissues of the positive and negative modulators. The next challenge will be to understand the cellular context in which the various positive and negative modulators participate in NFκB signaling and how these different control layers can be manipulated to treat specific inflammatory diseases without unwanted side effects.

## Materials and methods

### cDNA clone collection and construction

A set of bacterial stocks from the Mammalian Gene Collection (MGC) containing 30,720 cDNA clones in 80 384-well plates were ordered from the American Type Culture Collection (Manassas, VA). Putative full-length mouse and human cDNA clones in mammalian expression vectors were identified by cross-referencing the library with the 'Static Clone List' generated by MGC [53] and the 'IRAK/IRAL Cumulative File' generated by the IMAGE consortium [54] from which the MGC set is derived. Full-length status was determined by the MGC and IMAGE consortium through end sequencing, and full-length clones were given a GenBank identifier. The bacterial stocks for the cDNA clones matching the full-length criteria and cloned in mammalian expression vectors were rearrayed using a Genetix Q-bot (Hampshire, UK) into 96-well plates. A total of 8,700 full-length mouse and 5,800 full-length human genes were rearrayed. The rearrayed bacterial clones were amplified using a Hi-Gro incubator (Genomic Solutions, Ann Arbor, MI) in deep-well, 96-well plates containing 1.5 ml of Terrific Broth plus antibiotic (20 h; 500 rpm). Plasmid DNA was isolated from the bacterial clones and purified using a Roboprep 2500 (MWG, High Point, NC) and Macherey-Nagel NucleoSpin Robot-96 kits (Macherey-Nagel, Easton, PA). After purification, DNA concentrations were quantified on the basis of absorbance at 260 nm and normalized to a standard concentration of 2 ng/μl using a Theonyx liquid-handling robot (MWG). Individual clones were sequenced at random to verify gene inserts using universal M13 sequencing primers.

### Cell culture and plasmids

Human embryonic kidney (HEK)-293T cells were obtained from the American Type Culture Collection (ATCC No. CRL-11268, Rockville, MD) and were grown in Dulbecco's Modified Eagle Medium supplemented with 10% fetal bovine serum and 100 U/ml penicillin-streptomycin (Invitrogen, Carlsbad, CA). Cells were maintained at 37°C with 5% CO_2 _in a humidified chamber. The plasmids used in this study were from the following sources: pNFκB-Luc (5X TGGGGACTTTCCGC) and pGRE-Luc (Stratagene, La Jolla, CA); pCMV-Sport6-Empty and pUC18-Empty (Invitrogen); pCS2-DsRed (kindly provided by R. Davis); pCMX-IKBKB SS-AA (Ser177A & Ser181A) and pCMX-IKBKB SS-EE (Ser177E & Ser181E) (kindly provided by S. Chanda); pCMV-MAP3K7 (K63W) (kindly provided by J. Ninomiya-Tsuji); pCMV-ΔTRAF2 (1-211) (kindly provided by G. Natoli); and pcDNA3-HA-IKBKG (134-419) (kindly provided by G. Nuñez).

### High-throughput transfection and assay conditions

High-throughput transfections for the gain-of-function screens were performed in 384-well plates with each well containing 20 μl OptiMEM with GlutaMax (no serum or antibiotic), 20 ng pNFκB-Luc, 15 ng pCS2-DsRed, and 150 nl Fugene6 (Roche Diagnostics, Indianapolis, IN). The transfection mix was dispensed using a Multidrop-384 (Titertek, Huntsville, AL). Purified plasmid DNA (15 ng) for each gene was added to an individual well of a 384-well plate using a Biomek FX robot (Beckman Coulter, Fullerton, CA) and the DNA:Fugene mixture was incubated for 30 min at room temperature. Each 384-well plate contained eight empty wells for the manual addition of positive and negative control plasmids. A pCMV-Sport6-Empty plasmid lacking a cDNA insert was included in each plate as the negative control. Following incubation, HEK-293T cells were added to each well in 20 μl serum-containing medium (20% fetal bovine serum, 200 U/ml penicillin-streptomycin) at a concentration of 400,000 cells/ml. The plates were then incubated for 48 h at 37°C with 5% CO_2_. At the end of the incubation period, 40 μl BriteLite (Perkin Elmer, Wellesley, MA) was added to each well using the Multidrop-384 (Titertek). Within 5 min after substrate addition, luminescence and fluorescence were read using an Analyst HT plate reader (Molecular Devices, Sunnyvale, CA). For the loss-of-function screens, transfections were performed identically to the gain-of-function screens except that either purified TNF or IL1B (Sigma Aldrich, St. Louis, MO; Catalog Nos. T0157 and I9401) was added in 10 μl medium, 4 h post-transfection. The concentrations of TNF and IL1B were 20 ng/ml (final) and 0.5 ng/ml (final), respectively.

To contextually organize the positive NFκB modulators, each combinatorial screen was run using paired 384-well plates. In one of the paired plates, the positive modulators were co-transfected with either 15 or 20 ng of the dominant-negative expression plasmid. Fifteen ng of the dominant-negative expression plasmid was used for pCMV-ΔTRAF2 and pCMV-MAP3K7 (K63W) while 20 ng was used for pCMX-IKBKB SS-AA and pcDNA3-HA-IKBKG (134-419). In the second paired plate, the positive modulators were co-transfected with either 15 ng or 20 ng of pUC18-Empty to match the amount of the dominant-negative expression plasmid. The pUC18-Empty was used as a control for the dominant-negative mutant to keep the total amount of DNA constant. Both of the paired 384-well plates contained eight empty wells for manual addition of positive and negative control plasmids. A pCMV-Sport6-Empty plasmid was included on each paired plate as the negative control for the positive modulator. Assay conditions were identical to those described for the gain-of-function screen except that the amount of Fugene6 (Roche Diagnostics) was increased to either 195 nl (TRAF2, MAP3K7) or 210 nl (IKBKB, IKBKG). To contextually organize the negative NFκB modulators, paired 384-well plates were also used. In one of the paired plates, the negative modulator was co-transfected with 10 ng of the pCMX-IKBKB SS-EE plasmid expressing the IKBKB constitutively active mutant. In the second paired plate, the negative modulators were co-transfected with 10 ng pUC18-Empty. The pUC18-Empty was used as a control for the constitutively active mutant to keep the total amount of DNA constant. Both of the paired 384-well plates contained eight empty wells for manual addition of positive and negative control plasmids. A pCMV-Sport6-Empty plasmid was included on each paired plate as the negative control for the negative modulator. Assay conditions were identical to those described for the gain-of-function screen except that the amount of Fugene6 (Roche Diagnostics) was increased to 180 nl. Each paired combinatorial screen was performed at least three times.

### Analysis of the gain-of-function and loss-of-function screening data

Luciferase measurements for each gene in the gain-of-function and loss-of-function screens were adjusted for background luminescence using control wells containing only media and BriteLite (PerkinElmer). The background-subtracted values were then normalized for transfection efficiency using the fluorescent DsRed measurements. Normalized fold-change ratios were calculated by dividing the normalized luciferase values for each gene by the normalized luciferase values for the pCMV-Sport6-Empty negative control on each plate. In the case where multiple control wells on each plate were transfected with the pCMV-Sport6-Empty plasmid, the average normalized luminescence value was used to calculate the normalized fold-change ratio. In the initial gain-of-function screen, significant positive modulators were required to have a normalized fold-change ratio greater than three-fold and luminescence values greater than 5 standard deviations (SD) from the experimental mean. In the initial loss-of-function screen, significant negative modulators were required to have a normalized fold-change ratio less than 0.34 (that is, > 3-fold reduced) and luminescence values less than 5 SD from the experimental mean. Confirmation assays on the significant positive and negative modulators were performed at least three times and analyzed using a one-sample *t*-test for its statistical significance from a fold change of 1. Probability values were adjusted for multiple comparisons using a false-discovery rate correction [55]. Confirmed hits were required to have an average normalized fold-change ratio greater than three-fold (positive modulators) or less than 0.34 (negative modulators) and an adjusted *p *value < 0.05.

Functional analysis of the positive and negative modulators was performed using NIH David 2.1 [56] and based on molecular function ontologies at level 2. Mapping of positive and negative modulators to the known signaling pathways was performed using GenMAPP 2.1 [57]. The canonical TNF, IL1, B-cell receptor, and T-cell receptor signaling pathways were obtained from the Human Protein Reference Database [58].

### Analysis of tissue expression

Gene-expression data for 79 human tissues using the human Affymetrix U133A array were obtained from Symatlas [23]. The raw data were preprocessed using GCRMA with a log_2 _transformation. The mean expression level and a 99% confidence interval for each gene were then calculated across all 79 human tissues. Using the lower 99% confidence limit as a cutoff, modulators that fell below the cutoff were considered absent in that tissue, whereas those that were expressed above the cutoff were considered present. Statistical analysis of the average number of modulators present in immune tissue versus non-immune tissue was performed using a two-sample *t*-test.

### Analysis of the combinatorial screens to contextually organize positive and negative modulators

Combinatorial screens for the positive modulators were analyzed by first subtracting background luminescence values and normalizing for transfection efficiency using the fluorescent DsRed measurements. Normalized fold-change ratios were calculated for each positive modulator on each of the paired 384-well plates by dividing the normalized luciferase values by the normalized luciferase values for the pCMV-Sport6-Empty negative control. A percent reduction was calculated based on the normalized fold-change ratio of the positive modulator that was co-transfected with the dominant-negative mutant and the normalized ratio of the positive modulator that was co-transfected with pUC18-Empty. The percentage reduction by the dominant-negative mutant was averaged for at least three experimental replicates and then subjected to a lower-bound cutoff that flagged each positive modulator as either upstream or unclassified relative to the dominant-negative mutant. A lower-bound cutoff of ≥ 70% was used to flag positive modulators as upstream. Percent reductions less than the cutoff were flagged as unclassified. Analysis of the combinatorial screens for the negative modulators was performed similarly, except that modulators showing ≥ 70% reduction were flagged as downstream relative to the constitutively active mutant and all others were flagged as unclassified.

### Signaling network construction

An adjacency matrix was constructed based on the orientation flags (that is, upstream, downstream, or unclassified) from the dominant-negative and constitutively active screens used in the contextual organization analysis. The adjacency matrix containing the experimental data was then merged with a separate adjacency matrix containing the known NFκB signaling network. On the basis of the combined adjacency matrix a signaling network map was constructed as a rooted tree with the NFκB complex serving as the obligate root node. The longest path was calculated from each terminal node to the root node using a previously defined algorithm [47]. For example, if A is upstream of B and C, and B is upstream of C but downstream of A, then the graph would be drawn as A → B → C and not as A → B, A → C and B → C. The signaling networks were visualized using Cytoscape software [59].

## Additional data files

The following additional data are available online with this paper. Additional data file 1 is a table containing a complete list of positive NFκB modulators identified using the gain-of-function full-length gene screens. Additional data file 2 is a table containing a complete list of negative NFκB modulators identified using the loss-of-function full-length gene screens. Additional data file 3 is a table listing the reduction in NFκB activation by the positive modulators using the IKBKB, IKBKG, MAP3K7, and TRAF2 dominant-negative mutants. Additional data file 4 is a table listing the reduction in NFκB activation by a constitutively active IKBKB mutant using the negative NFκB modulators. Additional data file 5 is a figure of the overlap and location of positive and negative modulators identified in the cell-based screens within the canonical TNF (A) and IL1 (B) signaling pathways. The signaling pathways were obtained from the Human Protein Reference Database and visualized using GenMAPP 2.1. The green shaded boxes represent positive modulators and red shaded boxes represent negative modulators. Green shaded boxes with red borders represent modulators that were identified as both positive and negative. Additional data file 6 is a figure showing the overlap and location of positive and negative modulators identified in the cell-based screens within the canonical B-cell receptor (A) and T-cell receptor (B) signaling pathways. The signaling pathways were obtained from the Human Protein Reference Database and visualized using GenMAPP 2.1. The green shaded boxes represent positive modulators and red shaded boxes represent negative modulators. Green shaded boxes with red borders represent modulators that were identified as both positive and negative. Additional data file 7 is a figure showing a network map of the NFκB modulators. An adjacency matrix was constructed on the basis of the orientation flags from the dominant-negative and constitutively active screens (that is, upstream, downstream, or unclassified). The adjacency matrix containing the experimental data was merged with a separate adjacency matrix containing members of the currently accepted NFκB signaling network. On the basis of the combined adjacency matrix, a network map was constructed as a rooted tree with the NFκB complex serving as the obligate root node. The longest path was calculated from each terminal node to the root node. Peach nodes = positive modulators; green nodes = negative modulators; pink nodes = genes identified as both positive and negative modulators; orange nodes = members of the known NFκB signaling network; red nodes = dominant-negative mutants (plus TRAF2); blue nodes = positive modulators whose network location was adjusted based on the currently accepted NFκB network structure.

## Supplementary Material

Additional data file 1 A complete list of positive NFκB modulators identified using the gain-of-function full-length gene screens. Click here for file

Additional data file 2 A complete list of negative NFκB modulators identified using the loss-of-function full-length gene screens. Click here for file

Additional data file 3 A table listing the reduction in NFκB activation by the positive modulators using the IKBKB, IKBKG, MAP3K7, and TRAF2 dominant-negative mutants. Click here for file

Additional data file 4 A table listing the reduction in NFκB activation by a constitutively active IKBKB mutant using the negative NFκB modulators. Click here for file

Additional data file 5 A figure of the overlap and location of positive and negative modulators identified in the cell-based screens within the canonical TNF (A) and IL1 (B) signaling pathways. The signaling pathways were obtained from the Human Protein Reference Database and visualized using GenMAPP 2.1. The green shaded boxes represent positive modulators and red shaded boxes represent negative modulators. Green shaded boxes with red borders represent modulators that were identified as both positive and negative. Click here for file

Additional data file 6 A figure showing the overlap and location of positive and negative modulators identified in the cell-based screens within the canonical B-cell receptor (A) and T-cell receptor (B) signaling pathways. The signaling pathways were obtained from the Human Protein Reference Database and visualized using GenMAPP 2.1. The green shaded boxes represent positive modulators and red shaded boxes represent negative modulators. Green shaded boxes with red borders represent modulators that were identified as both positive and negative. Click here for file

Additional data file 7 A figure showing a network map of the NFκB modulators. An adjacency matrix was constructed on the basis of the orientation flags from the dominant-negative and constitutively active screens (that is, upstream, downstream, or unclassified). The adjacency matrix containing the experimental data was merged with a separate adjacency matrix containing members of the currently accepted NFκB signaling network. Based on the combined adjacency matrix, a network map was constructed as a rooted tree with the NFκB complex serving as the obligate root node. The longest path was calculated from each terminal node to the root node. Peach nodes = positive modulators; green nodes = negative modulators; pink nodes = genes identified as both positive and negative modulators; orange nodes = members of the known NFκB signaling network; red nodes = dominant-negative mutants (plus TRAF2); blue nodes = positive modulators whose network location was adjusted based on the currently accepted NFκB network structure. Click here for file
