# Supplementary figures and images for: A functional map of NFκB signaling identifies novel modulators and multiple system controls
Source: Genome Biol. 2007 Jun 6;8(6):R104. doi: 10.1186/gb-2007-8-6-r104 (PMC2394752; doi:10.1186/gb-2007-8-6-r104)

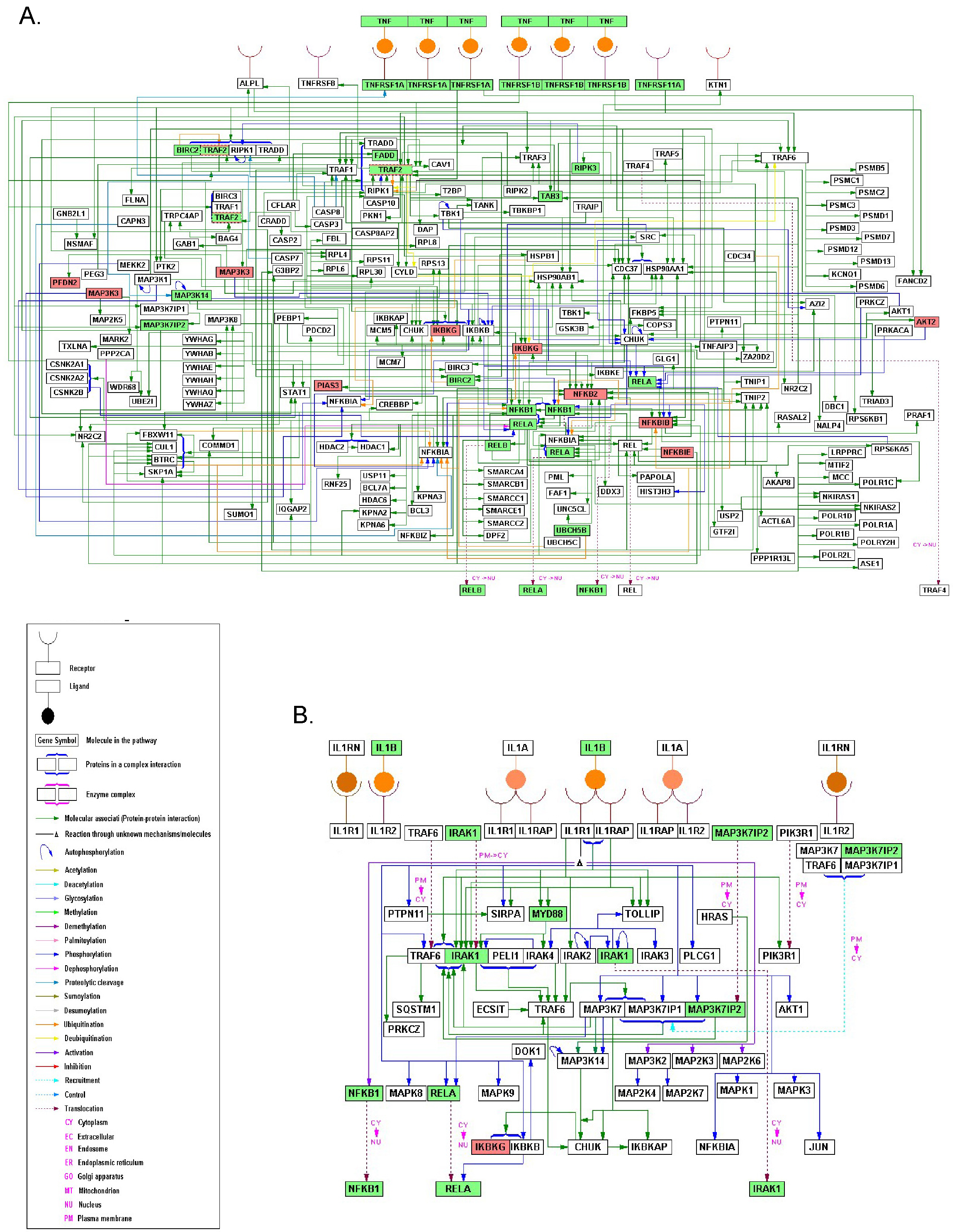

Supplement: Additional data file 5 — A figure of the overlap and location of positive and negative modulators identified in the cell-based screens within the canonical TNF (A) and IL1 (B) signaling pathways. The signaling pathways were obtained from the Human Protein Reference Database and visualized using GenMAPP 2.1. The green shaded boxes represent positive modulators and red shaded boxes represent negative modulators. Green shaded boxes with red borders represent modulators that were identified as both positive and negative. [file gb-2007-8-6-r104-S5.jpeg]

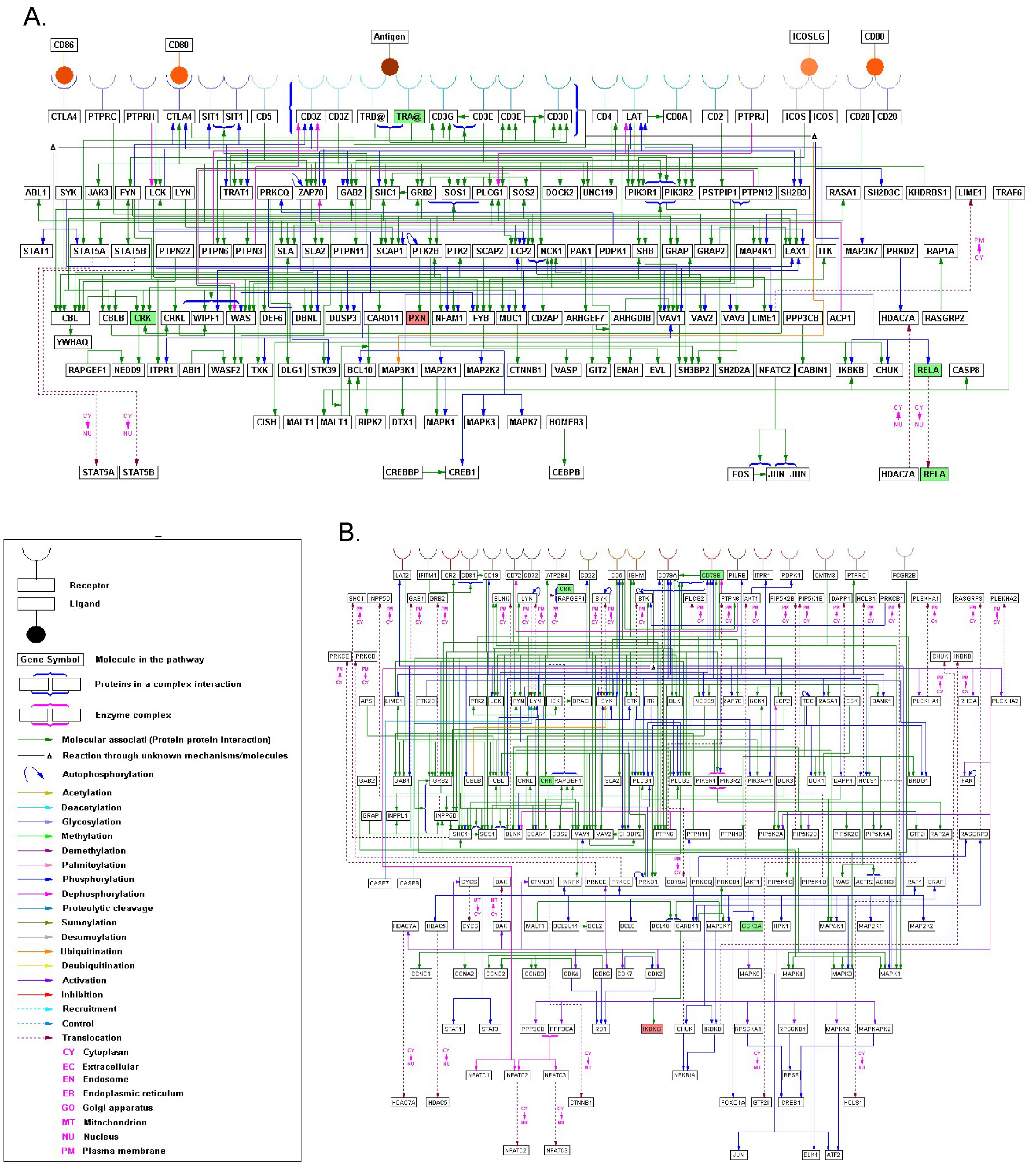

Supplement: Additional data file 6 — A figure showing the overlap and location of positive and negative modulators identified in the cell-based screens within the canonical B-cell receptor (A) and T-cell receptor (B) signaling pathways. The signaling pathways were obtained from the Human Protein Reference Database and visualized using GenMAPP 2.1. The green shaded boxes represent positive modulators and red shaded boxes represent negative modulators. Green shaded boxes with red borders represent modulators that were identified as both positive and negative. [file gb-2007-8-6-r104-S6.jpeg]

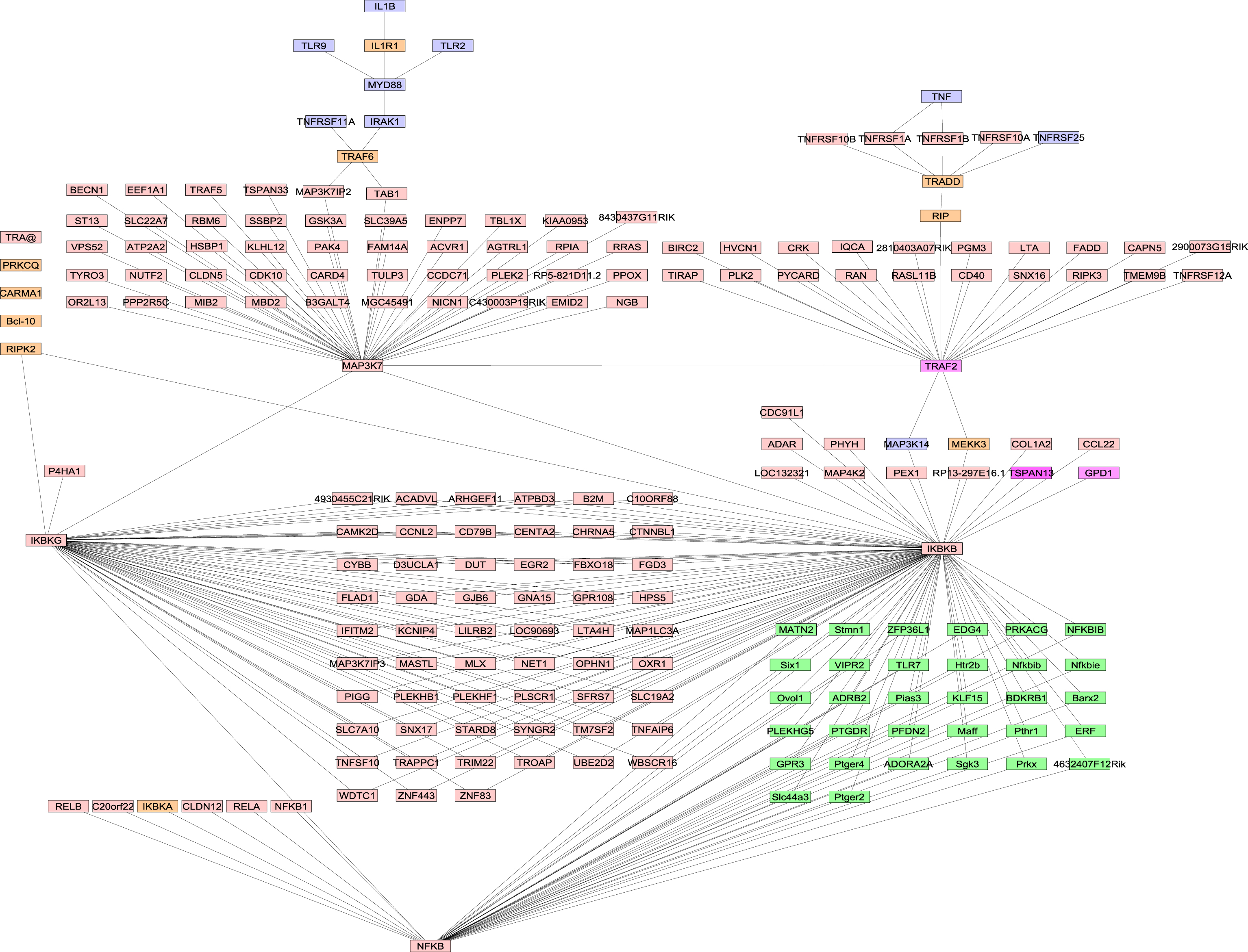

Supplement: Additional data file 7 — A figure showing a network map of the NFκB modulators. An adjacency matrix was constructed on the basis of the orientation flags from the dominant-negative and constitutively active screens (that is, upstream, downstream, or unclassified). The adjacency matrix containing the experimental data was merged with a separate adjacency matrix containing members of the currently accepted NFκB signaling network. Based on the combined adjacency matrix, a network map was constructed as a rooted tree with the NFκB complex serving as the obligate root node. The longest path was calculated from each terminal node to the root node. Peach nodes = positive modulators; green nodes = negative modulators; pink nodes = genes identified as both positive and negative modulators; orange nodes = members of the known NFκB signaling network; red nodes = dominant-negative mutants (plus TRAF2); blue nodes = positive modulators whose network location was adjusted based on the currently accepted NFκB network structure. [file gb-2007-8-6-r104-S7.jpeg]
